# Nitrite-induced testicular toxicity in rats: therapeutic potential of walnut oil

**DOI:** 10.5935/1518-0557.20180062

**Published:** 2019

**Authors:** Sunday A. Adelakun, Victor O. Ukwenya, Babatunde S. Ogunlade, Julius A. Aniah, Ayooluwa G. Ibiayo

**Affiliations:** 1 Department of Human Anatomy, School of Health and Health Technology, Federal University of Technology, Akure, Nigeria; 2 Department of Anatomy, Ladoke Akintola University of Technology, Ogbomosho, Oyo State, Nigeria; 3 Department of Anatomy, College of Medicine, University of Abuja, Federal Capital Territory (FCT), Nigeria; 4 Department of Anatomy College of Medicine, University of Lagos, Lagos, Nigeria

**Keywords:** spermatogenesis, nitrite, testis, Hormone, oxidative stress

## Abstract

**Objective:**

To determine the impact of walnut oil on nitrite-induced testicular toxicity in Sprague-Dawley (SD) rats. Available evidence suggests that walnut oil contains high levels of important unsaturated fatty acids including alpha-linolenic acid (ALA) and omega-3; nitrite is a reproductive toxicant that causes the loss of germ cells in the seminiferous tubules and generates oxidative stress in the testes, thus reducing sperm counts and affecting sperm morphology.

**Methods:**

This study included 24 male and 24 female adult SD rats. The male rats randomly assigned to Group A (controls) were given normal saline 2 ml/kg. The rats in Groups B, C, and D were given 50mg/kg body weight (bwt) of walnut oil, 0.08 mg/kg bwt of nitrite, and 0.08 mg/kg bwt of nitrite + 50 mg/kg of walnut oil respectively for 28 days via gastric gavage. Tested parameters included: testicular histology, sperm parameters, reproductive hormones, fertility, malondialdehyde (MDA), superoxide dismutase (SOD), reduced glutathione, and catalase (CAT).

**Results:**

A severe decrease in spermatogenic cell series, hypocellularity, tubular atrophy, decreased sperm quality, and increased MDA levels were observed in the rats given nitrite only when compared to controls. Rats given 50 mg/kg of walnut oil had significant growth of seminiferous epithelium compared to controls. The rats given walnut oil and nitrite had significant growth of seminiferous epithelium, improved sperm quality, and had decreased MDA levels.

**Conclusion:**

Walnut oil attenuated the deleterious effects of nitrite to the testes, reduced oxidative stress, and promoted spermatogenesis.

## INTRODUCTION

Walnut oil is a good source of omega-3 fatty acids that are essential for human nutrition (Iwamoto *et al.,* 2002). The major fatty acids found in walnut oil are linoleic, oleic, and linolenic acid (Tsamouris *et al.,* 2002). The preventive roles of monounsaturated fatty acids and polycyclic unsaturated fatty acids (MUFA and PUFA) in cardiovascular diseases have been identified (Tavakoli *et al.,* 2005). It has been reported that the consumption of walnut (kernel and oil) lowers blood cholesterol levels (Damasceno *et al.,* 2011; Rajaram *et al.,* 2009). Studies have shown that walnut oil has antioxidant properties and reduces the risk of coronary heart disease and inflammation, in addition to being useful in the treatment of skin diseases and high blood pressure (Labuckas *et al*., 2008; Fladman, 2002; Reiter *et al.,* 2005). Walnut is also effective in the treatment of type-2 diabetes and enhances cardiovascular flexibility (Tapsell *et al.,* 2009). It has been reported that due to its high concentration of natural antioxidants, walnut can be consumed as a protection against certain types of cancer (Bostani *et al.,* 2014). It may also reduce the risk of cardiovascular disease (Miraliakbari & Shahidi, 2008; Yang *et al*., 2009).

Nitrate is one of the most common contaminants in rural and suburban areas due to its high solubility in water; the contamination of ground water by nitrate originates primarily from fertilizers, septic systems, and manure storage or spreading operations. (McCasland *et al.,* 1985). Nitrite is a normal component of human diet found in most vegetables (Dennis & Wilson, 2003). Spinach and lettuce may contain as much as 2500 mg/kg of the compound, followed by 302.0mg/kg in curly kale, 61.0mg/kg in green cauliflower, and 13mg/kg in asparagus. Nitrite levels in 34 vegetable samples including different varieties of cabbage, lettuce, spinach, parsley, and turnips ranged between 1.1 and 57mg/kg (Correia *et al.,* 2010; Leszczyńska *et al.,* 2009). Fresh meat contains 0.4-0.5mg/kg of nitrite and 4-7mg/kg of nitrate (Dennis & Wilson, 2003). The presence of nitrite in animal tissue is a consequence of the metabolism of nitric oxide (Meulemans & Delsenne, 1994). Nitrite can be reduced to nitric oxide or ammonia by many species of bacteria (Cymeryng *et al.,* 1998). Under hypoxic conditions, nitrite may release nitric oxide, which causes potent vasodilation. Nitrate (NO_3_), and its chemical cousin Nitrite (NO_2_), can cause methemoglobinemia or blue baby syndrome (Kostić *et al.,* 1998). High nitrate levels may also indicate the presence of other pollutants, such as bacteria or pesticides, as these may follow the same path as nitrate into the water supply (McCasland *et al.,* 1985). It has been reported that inorganic nitrate and inorganic nitrite inhibited steroidogenesis in mouse Leydig tumor cells (MLTC-1) (Panesar, 1999; Panesar & Chan, 2000). Both nitrite and nitrate can endogenously be converted to nitric oxide (NO) (Ellis *et al.,* 1998). The inhibitory effects of nitrate and nitrite occur through the action of the metabolite nitric oxide (NO), which is an inhibitor of steroid hormone synthesis (Masuda *et al.,* 1997; Natarajan *et al.,* 1997).

The present study focused on the salutary role of walnut oil in nitrite-induced testicular toxicity in Sprague-Dawley rats.

## MATERIALS AND METHODS

### Collection of plants and preparation of walnut oil

Walnuts were collected from Igbara oke, Ondo State, Nigeria and were identified and authenticated in the Department of Agronomy, Ladoke Akintola University of Technology, Ogbomoso, Nigeria; a voucher specimen of the plant was deposited for future reference. After cleaning and drying in the shade, the air-dried walnut kernels were weighed using a CAMRY (EK5055, Indian) electronic scale and milled in an automatic electrical blender (model FS-323, China) to powdered form. Then, a portion of powdered walnut was kept in solvent n-hexane in the laboratory; after 24 hours in the solvent, the sample was strained and the solution obtained was poured into a rotary device (Rotavapor^®^ model ED-100) at 40-50ºC to let the solvent evaporate. To ensure the removal of moisture, the samples were kept in a vacuum desiccator (GCD-064X, KIKO, Japan) for an additional 24 hours. At the end of the process, a bright yellow oily substance with a density of 1.1485 gr/ml was obtained.

### Animal care and experimental design

A total of 24 male and 24 female adult Sprague-Dawley rats were used in this study. Twenty-four male Sprague-Dawley rats of the first filial generation were randomly assigned to three treatment groups identified as B, C and D or the control group A (n=6 in each group). Orogastric tubes were used to administer the following to the animals in treatment groups B, C and D, respectively: 0.08 mg/kg body weight nitrite; 50mg/kg body weight walnut oil and 0.08mg/kg body weight of nitrite; and 50mg/kg body weight of walnut oil for 28 days. The animals in the control group (group A) were administered equal amounts of phosphate buffered saline (PBS). All the animals were housed in clean, well-ventilated cages measuring 34.0x20.5x20.0cm (temperature: 28-31°C; humidity: 50-55%) (Yakubu *et al.,* 2008). The cages were cleaned daily. All animals were checked for illnesses, abnormal behavior, and morphological anomalies. All experimental procedures followed the recommendations provided in the “Guide for the Care and Use of Laboratory Animals” prepared by the National Academy of Sciences and published by the National Institute of Health (NIH, 1985). The rats were fed with standard chow at a recommended daily dose of 100 g/kg as advised by the International Centre of Diarrheal Disease Research, Bangladesh (ICDDR, B). Drinking water was supplied *ad libitum*. The weights of the rats were documented at procurement, during the period of acclimatization, at commencement of administrations, and once a week throughout the period of the experiment, using a CAMRY electronic scale (EK5055, Indian).

### Surgical procedure

Twenty-four hours after the last administration, the rats were given intraperitoneal pentobarbital sodium (40mg/kg) and their peritoneal cavities were opened through a lower transverse abdominal incision. Then the testes of the rats in the control and experimental groups were immediately removed. The weights of the testes from each group were recorded. The animals were decapitated between 9:00 and 11:00 AM, and blood samples were collected. The blood samples were centrifuged at 4ºC for 10 min at 250×g and the serum obtained was stored at −20ºC until assayed. The harvested testis specimens were fixed in Bouin’s fluid for histological analysis (Avwioro, 2010).

### Epididymis sperm count, viability and motility

Spermatozoa from the cauda epididymis were released by cutting into 2ml of medium (Hams F10) containing 0.5% bovine serum albumin (Feng *et al.,* 2001). After 5 min of incubation at 37ºC (with 5% CO_2_), the cauda epididymis sperm reserves were determined using a hemocytometer. Sperm motility was analyzed with a microscope (Leica DM750) and reported as the mean number of motile sperm according to the method developed by the WHO (WHO, 1999).

### Biochemical estimations

Lipid peroxidation products were estimated by measuring TBARS and were determined in accordance with the method published by Niehaus & Samuelsson (1968). Nonenzymatic antioxidants such as reduced glutathione (GSH) and catalase (CAT) were estimated as described by Ellman (1959) and Sinha (1972), respectively. SOD activity in the testes was determined according to the method described by Marklund & Marklund (1974)

### Hormone determination

The hormonal profiles of endocrine markers testosterone (TT), follicule stimulating hormone (FSH) and leutenizing hormone (LH) were measured using commercially available immunoassay (ELISA) kits (Randox Laboratories Ltd, Admore Diamond Road, Crumlin, Co., Antrim, United Kingdom, Qt94QY) according to manufacturer instructions.

### Testicular histology preparation

The testes were harvested and fixed in Bouin‘s fluid for 24h and were then transferred to 70% alcohol for dehydration. The tissues were passed through 90% and absolute alcohol and xylene for different durations before they were transferred into two changes of molten paraffin wax for 1 hour each in an oven at 65◦C for infiltration. They were subsequently embedded and serial sections cut on a rotary microtome set at 5 microns. The tissues were picked up with albumenized slides and allowed to dry on hot plates for 2 min. The slides were dewaxed with xylene and passed through absolute alcohol (two changes), 70% alcohol, 50% alcohol, and then water for 5 min. The slides were then stained with Hematoxylin and Eosin. The slides were mounted in DPX. Photomicrographs were taken at a magnification of 100x on a Leica DM750 microscope.

### Morphometric studies

Morphometric studies were carried out as reported by Akang *et al.* (2015). The primary aim was to estimate the volumes of seminiferous tubule epithelium (seminiferous epithelium) and interstitium in the testes. This was done in accordance with Howard & Reed (2005) and Baines *et al*. (2008). Four sections per testis and six microscope fields per section were randomly chosen for analysis. Fields were sampled as images captured on a Leica DM750 bright field microscope (Germany) via LAZ software. Volume densities of testicular ingredients were determined by randomly superimposing a transparent grid comprising 35 test points arranged in a quadratic array. Test points falling on a given testis and its ingredients were summed over all fields from all sections. The total number of points hitting on a given ingredient (lumen (EL), epithelium (EE), interstitium (EI)), divided by the total number of points hitting on the testis sections (ET) multiplied by 100, provided an unbiased estimate of its percent volume density/volume fraction.

### Fertility Testing

Fertility testing was performed by a modification of the method reported by Ligha *et al.* (2012). Each male rat was isolated, placed in a cage and paired with a pro-estrous female rat in the first hours of the estrous cycle as determined by vaginal smear examination. On the following day, the female rats were checked after mating to detect spermatozoa in their vagina by microscopic examination of the vaginal fluid. Females in which sperm plugs were detected the following morning after mating were assumed to be on day one of gestation. The fetuses were removed by ventral laparotomy on the 21^st^ day of gestation and counted.

### Data presentation and statistical analysis

Data were expressed as Mean±SEM. Statistical differences between the groups were evaluated by one-way ANOVA, followed by Dunnett’s comparison test to compare between treated and control groups. Differences yielding *p*<0.05 were considered statistically significant. Statistical analyses of data were performed using GraphPad Prism 5 Windows (GraphPad Software, San Diego, California, USA).

## RESULTS

### Testes and body weight

The results are listed in Table 1. There was a significant decrease in the mean testis weight in the nitrite treated group compared with controls (*p*<0.05). There were no significant differences in testis weight between the other groups. The bodyweight of the rats given 50 mg/kg body weight of walnut increased (242.3±6.75) when compared to controls (237.3±7.25), while the body weight of the rats given nitrite (181.7±7.91) and nitrite + walnut oil (212.0±6.25) decreased significantly (*P*<0.05). The body weight of the rats in the walnut oil group (242.3±6.75) were significantly different (*p*<0.05) from the body weight of the rats given nitrite (181.7±7.91) and nitrite + walnut oil (212.0±6.25).

**Table 1:** Effect of walnut oil on testis weight, body weight, and sperm parameters of nitrite treated rats.

Parameters	Groups			
A (control)	B (50 mg/kg bwt walnut oil)	C (0.08 mg/kg bwt Nitrite)	D (0.08 Nitrite + 50 walnut oil) mg/kg bwt
Testis weight (g)	1.52±0.05	1.45±0.07	1.03±0.04*	1.39±0.06
Body weight (g)	237.3±7.25	242.3±6.75 ^β^	181.7±7.91*^, α^	212.0±6.25*
Sperm count (x10^6^/ml)	75.58±3.56	72.69±3.17^β^	34.39±2.85*^, α^	66.54±3.53 ^β^
Motility (%)	70.04±4.95	73. 41±4.75 ^β^	28.19±3.28*^, α^	65.69±4.06 ^β^
Viability (%)	80.33±2.86	82.63±3.12 ^β^	51.86±3.36*^, α^	68.11±2.26 ^β^

Values are expressed as Mean ± S.E.M, n=6 in each group

* significantly different from the control group

β significantly different from the nitrite group

α significantly different from the walnut oil group *p*<0.05. One-Way ANOVA. bwt: body weight

*p*<0.05. One-Way ANOVA

bwt body weight

### Sperm motility, viability and count

Nitrite treatment significantly decreased sperm count, motility, and viability in the nitrite group compared with controls and other experimental groups. Sperm count, motility, and vitality were 34.39±2.85, 28.19±3.28 and 51.86±3.36, respectively, in the nitrite treated group. The corresponding values in the walnut oil group were 72.69±3.17, 73.41±4.75, and 82.63±3.12, and the corresponding values in the nitrite + walnut oil group were 66.54±3.53, 65.69±4.06, and 68.11±2.26. However, sperm count, motility, and vitality in controls were 75.58±3.56, 70.04±4.95, 80.33±2.86. There were no significant changes in the proportions of sperm abnormalities in the experimental groups compared with controls (Table 1).

### Antioxidant (SOD, GSH, CAT) and MDA levels

As shown in Table 2, MDA levels in the nitrite group increased when compared with the control group and decreased in the nitrite + walnut oil group in relation to the nitrite group. Antioxidant levels (SOD, GSH, and CAT) decreased significantly in the nitrite group (*p<0.05) when compared to controls; SOD, GSH and CAT levels in the nitrite + walnut oil group decreased (*p<0.05) when compared to controls.

**Table 2:** Effects of walnut oil on antioxidant levels and lipid peroxidation of nitrite treated rats.

Parameters	Groups			
A (control)	B (50 mg/kg bwt walnut oil)	C (0.08 mg/kg bwt Nitrite)	D (0.08 Nitrite + 50 walnut oil) mg/kg bwt
Malondialdehyde (MDA) (nmol/mg)	5.17±0.46	3.49±0.20*^,β^	8.15±0.55 *	3.05±0.36 *^,β;^
Super oxide dismutase (SOD), (u/mg protein)	12.08±0.75	13.03±0.81^β^	4.39±0.33*^, α^	11.16±0.57^β^
Glutathione peroxidase, (umol/mg protein)	9.64±1.04	8.75±0.97 ^β^	4.17±0.21*^, α^	7.91±0.89 ^β^
Catalase, (u/mg protein)	19.93±1.23	21.45±1.38^β^	11.19±0.87*^, α^	17.51±1.24 ^β^

Values are expressed as Mean ± S.E.M

n=6 in each group,

* significantly different from the control group

β significantly different from the nitrite group

α significantly different from the walnut oil group

*p*<0.05. One-Way ANOVA.

bwt body weight

### Hormonal assay

**Figure 1:**
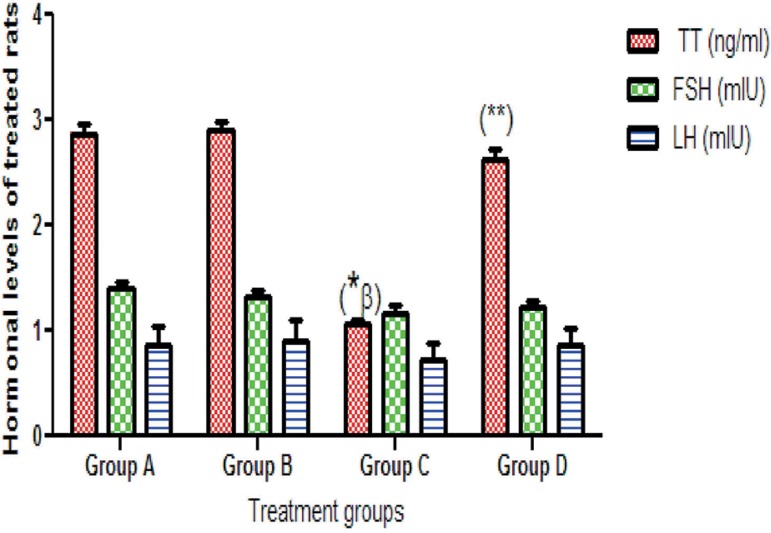
TT: Testosterone, FSH: Follicle stimulating hormone, LH: Luteinizing hormone A: Control B: 50 mg/kg bwt walnut oil, C: 0.08 mg/kg bwt Nitrite, D: (0.08 Nitrite + 50 walnut oil) mg/kg bwt; Values are expressed as Mean ± S.E.M n=6 in each group *:significantly different from the control group ** significantly different from the nitrite group β: significantly different from the walnut oil group *p*<0.05. One-Way ANOVA. bwt: bodyweight;

In comparison with controls (2.87±0.09), the rats in the nitrite group had significantly lower TT (1.06±0.05) (*p*<0.05) levels, while the LH and FSH (1.16±0.08 and 0.73±0.16) levels were not significantly changed (1.40±0.07 and 0.86±0.19). In the walnut oil and nitrite + walnut oil group, TT (2.91±0.08 and 2.63±0.10) as well as FSH (1.33±0.06 and 0.91±0.20) and LH (1.22±0.07 and 0.86±0.17) levels were not significantly different from the values seen in controls for TT (2.87±0.09), FSH (1.40± .07) and LH (0.86±0.19) (Fig. 1).

### Morphometric analysis

After the 28 days of the study, the volume density of germinal epithelium of controls (64.90±0.89) was significantly different from that of the rats given nitrite (58.08±0.33) (*p*<0.05). However, there was a significant increase in the walnut oil (69.18±0.37) and nitrite + walnut oil (70.66±0.22) groups when compared to controls (64.90±0.89) and rats given nitrite alone (58.08±0.33). Lumen density significantly decreased in the nitrite group (9.60±0.23) compared to the control group (15.42±0.20). The interstitium had a significant increase in the nitrite group (26.73±0.21) when compared to controls (21.06±0.26). Significant decreases were also seen in the interstitium of the rats in the walnut oil (16.91±0.27) and nitrite + walnut oil (20.39±0.17) groups compared to the nitrite group (26.73±0.21) (Table 3).

**Table 3:** Morphometric analysis of testes after 28 days of treatment.

Parameters	Groups			
A (control)	B (50 mg/kg bwt walnut oil)	C (0.08 mg/kg bwt Nitrite)	D (0.08 Nitrite + 50 walnut oil) mg/kg bwt
Germinal epithelium (%)	64.90±0.89	69.18±0.37*	58.08±0.33*^, α^	70.66±0.20*
Lumen (%)	15.42±0.20	12.52±0.18*	9.60±0.23*^, α^	11.99±0.12*
Interstitium (%)	21.06±0.26	16.91±0.27*^,β^	26.73±0.21*^, α^	20.39±0.71 ^β^

Values are expressed as Mean ± S.E.M n=6 in each group

* significantly different from the control group

β significantly different from the nitrite group

α significantly different from the walnut oil group *p* <0.05. One-Way ANOVA. bwt: body weight

*p*<0.05. One-Way ANOVA.

bwt body weight

### Testicular histology

Sections of the seminiferous tubules of control rats had moderately circular or oval outlines with normal stratified seminiferous epithelium showing cells of the spermatogenic series and spermatozoa within the lumen (Fig. 2A). The rats in the walnut oil group showed normal cellular composition in their germinal epithelium with sperm cells in the lumen and a normal interstitium and prominent Leydig cells (Fig. 2B). The seminiferous tubules of the rats treated with nitrite alone showed severe reduction of cells of the spermatogenic series, hypocellularity in the interstitium, widening of the tubular lumen, tubular atrophy, and fewer spermatozoa in the tubular lumen (Fig. 2C). The rats in the nitrite and walnut oil groups showed cells of the spermatogenic series and normal cellular composition in their germinal epithelium with sperm cells in the lumen and a normal interstitium (Fig. 2D).

**Figure 2:** Section of the testis of control rat showing the seminiferous tubules containing cells of the spermatogenic series (SS) and the lumen (L) containing spermatozoa; Black arrow represents spermatogonium; P represents primary spermatocytes; Blue arrow represents spermatids and spermatozoa. (A-B) Section of the testis of rat treated with nitrite showing hypocellularity, reduction in cells of the spermatogenic series (SS) as a result of degeneration, sloughing and shortening of seminiferous epithelium; The seminiferous tubules show a single layer of basal spermatogonia; widened empty lumen (L); widened interstitium (I) due to tubular atrophy as a result of degeneration, Leydig cells show hyperplasia (brown arrows) and V shows vascular hemorrhage. (C) Section of the testis of rat treated with nitrite and walnut oil showing cells of the spermatogenic series and normal cellular composition in their germinal epithelium with sperm cells in the lumen and a normal interstitium. (H&E; X100).


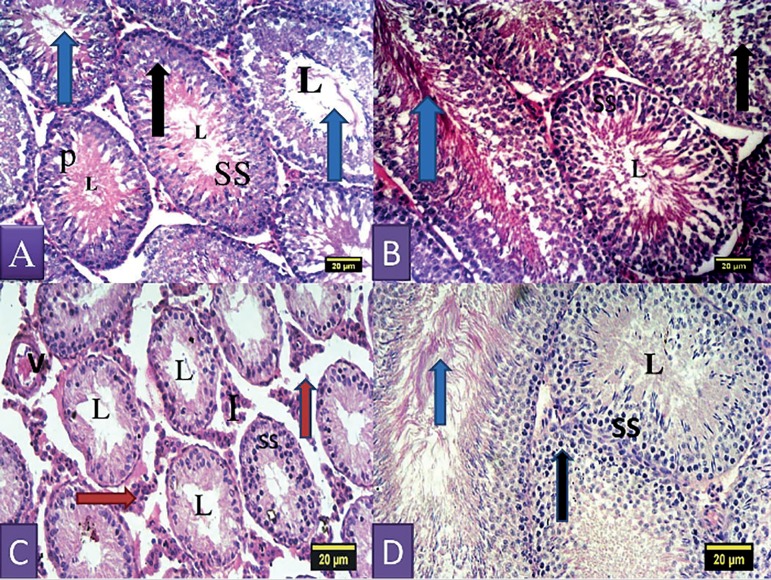


### Fertility testing in control and treated rats

The rats treated with 0.08mg/kg body weight of nitrite suffered with decreased fertility potential, as more than 90% of the female rats they mated with did not get pregnant. By their turn, the group given 0.08mg/kg body weight of nitrite and 50mg/kg body weight of walnut oil did not suffer such negative effects, since all the female rats they mated with got pregnant and had at least six fetuses. There was a decrease in the number of fetuses produced in group B treated with 50mg/kg body weight of walnut oil and 0.08mg/kg body weight of nitrite + 50mg/kg body weight of walnut oil when compared to controls. The number of pregnancies and fetuses was significantly decreased in group C rats given nitrite when compared with controls and rats in groups B and D (*p*<0.05) (Table 4).

**Table 4:** Fertility testing.

Parameters	Groups			
A (control)	B (50 mg/kg bwt walnut oil)	C (0.08 mg/kg bwt Nitrite)	D (0.08 Nitrite + 50 walnut oil) mg/kg bwt
No. of pregnancies	6.00±0.00	6.00±0.00	2.00±0.00	6.00±00
No. of fetuses	8.33±0.80	7.33±0.49^β^	0.67±0.56*	6.83±0.54 ^β^

Values are expressed as Mean ± S.E.M n=6 in each group

* significantly different from the control group

β significantly different from the nitrite group

α significantly different from the walnut oil group *p* <0.05. One-Way ANOVA. bwt: body weight

*p*<0.05. One-Way ANOVA.

bwt body weight

## DISCUSSION

Herbal remedies are alternative medications prepared from plants and plant extracts used to treat illnesses and diseases and to address psychological concerns. Herbal remedies have been around for centuries and are precursors to modern medicine (Burke *et al.,* 2006). Herbal remedies are obtained from a wide variety of natural sources including plant leaves, bark, berries, flowers, and roots (Khaki *et al.,* 2011). Walnut oil is reported to be a good source of omega-3 fatty acids that are essential for human nutrition (Özcan et al., 2010). In this study there was a great concern that exposure to nitrite might cause reproductive toxicity in rats. Our results showed that nitrite administration decreased the relative weights of the testes and the body weight of the included rats as reported in previous studies (Akintunde *et al*., 2014). At the end of our study, nitrite administration had significantly reduced sperm count, motility, and viability by subjecting the spermatozoa to increased oxidative stress-induced damage, since their plasma membranes contain large quantities of polyunsaturated fatty acids (PUFAs) (Bansal & Bilaspuri, 2010; Alvarez & Storey, 1995) and their cytoplasm contains low concentrations of scavenging enzymes (Saleh & Agarwal, 2002; Sharma & Agarwal, 1996). Increased formation of ROS has been correlated with decreased sperm motility (Aitken *et al.,* 1989; Armstrong *et al*., 1999).

The link between ROS and reduced motility may be due to a cascade of events that result in rapid loss of intracellular ATP leading to axonemal damage and sperm immobilization (Bansal & Bilaspuri, 2010; de Lamirande & Gagnon, 1995). Our investigation also demonstrated that exposure to nitrite decreased testosterone concentrations, indicating interference with steroidogenesis. Administration of walnut oil increased testosterone levels and indicated the positive effect of walnut oil on the hypothalamic-pituitary-testicular axis. The hypothalamic-pituitary-testicular axis can be affected by various negative and positive feedback mechanisms. Nitric oxide (NO) is one of the factors affecting this axis. High levels of arginine in walnut can be converted to nitric oxide. Nitric oxide increases the release of GnRH, which in turn increases gonadotropin secretion by activating the production of neuronal nitric oxide synthase in the pituitary gland (Barnes *et al.,* 2002; Pinilla *et al.,* 2001). Nitric oxide activates guanylate cyclase that causes the release of cyclic guanosine monophosphate, which by eventually raising GnRH, LH and FSH, enhances sperm motility and induces erection in males (Miraglia *et al.,* 2011). Co-treatment with walnut oil prevented damage to the testes from nitrite exposure. This indicates nitrite interferes with walnut oil-related metabolic functions. The competitive mechanism of interaction is a plausible mechanism of protection offered by walnut oil against nitrite toxicity.

This effect relates to the induction of oxidative stress. Our results showed that GPx, CAT, and SOD activities were distinctly lower in the testes of nitrite-exposed rats. Therefore, the increase in malondialdehyde (MDA), a by-product of lipid peroxidation (Dosumu *et al.,* 2012; Saleh & Agarwal, 2002), observed in the present study might be due to the concomitant increase in the generation of free radicals such as H_2_O_2_ and OH in the testes of the nitrite-treated rats. This depicts an increase in lipid peroxidation. The interaction between nitrite and essential trace elements might be one of the reasons for decreased levels of antioxidant enzymes in rat testes.

In this study, walnut oil increased testicular antioxidant enzymes and decreased MDA levels when administered alone. Walnut oil also prevented the ravaging effects of nitrite on sperm parameters and testicular antioxidant enzymes when administered with nitrite. It has been reported that walnut contains significant amounts of antioxidants, omega- 3 fatty acids and vitamin E, minerals, iron, sodium, calcium, magnesium, manganese, copper, potassium, phosphorus, protein, and fiber, which make it a varied nutritious meal (Cosmulescu *et al.,* 2009). Therefore, it is reasonable to infer that the antioxidant constituents of walnut boosted the testicular non-enzymatic and enzymatic antioxidants to effectively scavenge free radicals and thus prevent lipid peroxidation. The consequence is hereby reflected in increased sperm count and motility. This finding is in accordance with the reports of Ojobor *et al.* (2017). Moreover, vitamin E, a chain-breaking non-enzymatic antioxidant also found in walnut, inhibits lipid peroxidation in membranes by scavenging peroxyl (RO•) and alkoxyl (ROO•) radicals (Akang *et al.,* 2015; Saleh & Agarwal, 2002). The ability of vitamin E to maintain a steady state rate of peroxyl radical reduction in the plasma membrane depends on the recycling of vitamin E by external reducing agents such as ascorbate, found in walnut. The improved sperm parameters are also attributed to the abundant amount of vitamin E and zinc present in walnut oil, which are known male fertility agents as reported by Ajaiyeoba & Fadare (2006). Seeds of *T. conophorum* have been reported to contain reasonable amounts of zinc and vitamin E (Ojobor *et al.,* 2015). Furthermore, our study showed histological abnormalities in the testicular tissue of rats given nitrite such as sloughing and shortening of the seminiferous epithelium, which led to decreased counts of cells of the spermatogenic series. This is in agreement with a previous study by Akintunde *et al.* (2014), in which nitrite led to the sloughing off of germ cells in the seminiferous tubules and evident increases in histological lesions in the seminiferous tubules and epithelial lining of the testes among tested rats (Akintunde *et al*., 2010). Interstitial hyperplasia and absence of Sertoli cells in the seminiferous lumen concur with the study of Pant & Srivastava (2000), who reported effects on the histopathology of the testes of adult male mice after exposure to 900ppm potassium nitrate via drinking water in a study evaluating the endocrine disrupting effects of in-utero exposure to nitrate in rats. It has been clear for decades that testosterone produced in the interstitial cells of Leydig is a necessary prerequisite for the maintenance of established spermatogenesis (Zirkin, 1998). The reduced cellularity of the interstitium in the testes of the rats treated with nitrite alone might have produced a decrease in testosterone and consequently poor spermatogenesis.

Walnut oil also maintained the histological architecture of the testes, increased the proliferative activity of spermatogonia, and maintained cells of the spermatogenic series when compared to controls. Walnut has been reported to contain reasonable amounts of zinc and vitamin E (Ayoola *et al.,* 2011; Ojobor *et al.,* 2015), which decrease lipid peroxidation. From our findings, when walnut oil was co-administered with nitrite, it protected the testes from the harmful effects of nitrite. This protective nature of walnut is enhanced by some of its phytochemical constituents, namely zinc and vitamin E, known for protecting cell membranes and for their scavenging effects on free radicals. In clinical trials, vitamin E supplementation has been found to increase fertilization rates possibly by improving membrane integrity and decreasing oxidative damage and lipid peroxidation potential (Comhaire *et al.,* 2000; Geva *et al.,* 1996). We therefore inferred from our observations that the antioxidants and micronutrients in walnut militated against the ravaging effects of nitrite on the testes.

The results observed in the rats given nitrite plus walnut oil suggested that the administration of walnut oil at the dosages and times of treatment used in our study decreased the interference nitrite would otherwise have had in the development and maturation of the male gonad, as illustrated by the fact that all females mated with them got pregnant. The improvement of fertility in the group treated with nitrite plus walnut oil showed that walnut oil acts as a powerful antioxidant to protect the oxidative stress of nitrite on the testes. Walnut has been reported to contain zinc and vitamin E, and the latter has been described as an excellent lipid soluble chain-breaking antioxidant (Traber & Atkinson, 2007).

## CONCLUSION

In conclusion, we found that walnut oil effectively lowered nitrite-induced oxidative stress by reducing MDA levels and ameliorated the deleterious effects of nitrite on serum testosterone levels; however, it had no effect on serum FSH and LH levels, depleted the germinal epithelium, and caused hypocellularity and widening of the interstitium. Walnut oil did not only promote germinal epithelial growth, but also protected the cytoarchitecture of the testes from the damaging effects of nitrite. Walnut oil thus augmented spermatogenesis and defeated nitrite-induced oxidative stress through an antioxidant system of activities.
